# A substance use disorder training curriculum for internal medicine residents using resident-empaneled patients

**DOI:** 10.1186/s12909-024-05472-5

**Published:** 2024-04-30

**Authors:** Mim Ari, Julie L. Oyler

**Affiliations:** Department of Medicine, University of Chicago, 5841 S. Maryland Avenue, Chicago, IL 60637 USA

**Keywords:** Substance use disorder, Opioid use disorder, Alcohol use disorder, Curriculum development, Curriculum evaluation

## Abstract

**Background:**

Internal Medicine (IM) residents frequently encounter, but feel unprepared to diagnose and treat, patients with substance use disorders (SUD). This is compounded by negative regard for patients with SUD. Optimal education strategies are needed to empower IM residents to care for patients with SUD. The objective of this study was to evaluate a brief SUD curriculum for IM residents, using resident-empaneled patients as an engaging educational strategy.

**Methods:**

Following a needs assessment, a 2-part SUD curriculum was developed for IM residents at the University of Chicago during the 2018–2019 academic year as part of the ambulatory curriculum. During sessions on Opioid Use Disorder (OUD) and Alcohol Use Disorder (AUD), a facilitator covered concepts about screening, diagnosis, and treatment. In session, residents completed structured worksheets applying concepts to one of their primary care patients. A post-session assessment included questions on knowledge, preparedness & attitudes.

**Results:**

Resident needs assessment (*n* = 44/105, 42% response rate) showed 86% characterized instruction received during residency in SUD as none or too little, and residents did not feel prepared to treat SUD. Following the AUD session, all residents (*n* = 22) felt prepared to diagnose and treat AUD. After the OUD session, all residents (*n* = 19) felt prepared to diagnose, and 79% (*n* = 15) felt prepared to treat OUD. Residents planned to screen for SUD more or differently, initiate harm reduction strategies and increase consideration of pharmacotherapy.

**Conclusions:**

A brief curricular intervention for AUD and OUD using resident-empaneled patients can empower residents to integrate SUD diagnosis and management into practice.

**Supplementary Information:**

The online version contains supplementary material available at 10.1186/s12909-024-05472-5.

## Introduction

Given the high prevalence of substance use disorders (SUD), internal medicine (IM) residents frequently encounter patients with SUD [1]. Preparing a physician workforce to address SUD is crucial to improve care, a concept reinforced by the new 8-hour training requirement on management of opioid or other SUD for all Drug Enforcement Agency (DEA)-registered practitioners [2]. While psychiatry has historically been the “home” for addiction medicine, improving knowledge and confidence to treat SUD within Internal Medicine (IM) is necessary. Furthermore, exposure to SUD training during IM residency may help facilitate interest in advanced training, including Addiction Medicine fellowship, in this field. Accordingly, in 2022, the Accreditation Council of Graduate Medical Education (ACGME) added a requirement for Internal Medicine (IM) residencies to include clinical and educational experiences in addiction medicine (IV.C.2, IV.C.3), signaling this topic’s relevance for all internists [3, 4]. However, while IM residents frequently encounter patients with SUD, most feel unprepared to diagnose and treat SUD [5]. Compounding this lack of SUD knowledge and preparedness is stigma towards patients with SUD, with multiple studies showing lower “regard” for patients with addiction [6–8]. 

Wide variability exists in SUD content coverage in IM residencies with limited assessment of what, by what method, and how well this topic is being taught. For example, 72% of IM residencies reported opioid use disorder (OUD) didactics, but only 15% reported “very effective” teaching on this topic [9]. Optimal curricular strategies are needed to empower residents to integrate SUD diagnosis and management into their practices [10]. Previously published SUD curricula data has demonstrated increases in confidence, preparedness to diagnose and treat SUD, and responsibility to manage SUD [11–13], but most have required significant curricular time ranging from 6 to 16 sessions.

The objective of this study was to evaluate a brief SUD curriculum for IM residents, using resident-empaneled patients as an innovative and adaptable educational strategy. The goal of the overall curriculum is to empower residents with the knowledge and skills to care for patients with substance use disorder, ultimately leading to more compassionate and evidence-based care for patients with SUD.

## Methods

### Settings and participants

In 2018, all IM residents (*n* = 105, PGY1/2/3) at the University of Chicago were invited to complete an SUD curriculum needs assessment (Online Appendix 1). Based on the responses, a 2-part SUD curriculum was then developed for second and third year IM residents and delivered for the first time in the 2018–2019 academic year as part of the IM resident ambulatory curriculum. Participation in the ambulatory curriculum is expected for all second and third year IM residents (*n* = 60), but completing the post-session assessments was voluntary. The ambulatory sessions are variably attended due to other clinical requirements and vacation schedules. Some residents attended both sessions, some attended only one and some attended neither.

#### Program description

Using Kern’s six step approach for curriculum development and Vygotsky’s conceptual framework of situated learning-guided participation, 2 1-hour sessions were developed on Opioid Use Disorder (OUD) and Alcohol Use Disorder (AUD) [14, 15]. Session objectives are listed in the accompanying Table 1 and were similar in format and style. Residents were asked to choose a patient from their outpatient panel with the corresponding SUD. A patient case was provided if the resident did not choose one of their empaneled patients. During each session, the facilitator, an IM faculty member with experience in addiction medicine (author: MA), presented material covering key concepts about screening, diagnosis, and treatment of either OUD or AUD. This was interwoven with working individually through the sections of a structured worksheet related to the patient case (Online Appendix 2, 3). On their individual electronic devices, residents could access the electronic health record during the session to review information about the patient’s history, past clinic visits, medications and urine toxicology results. The worksheet served to reinforce and apply presented concepts culminating in creating an action plan for the patient, which the resident was encouraged to apply to the patient’s care at their next visit. The sessions were each delivered three times, as residents are assigned to one of three firms, and each firm had both an OUD and AUD session. The OUD and AUD sessions were delivered approximately two months apart.

**Table 1:** SUD curriculum goals & objectives

**SUD Curriculum Goal**:	
Empower residents with the knowledge and skills to care for patients with substance use disorder, ultimately leading to more compassionate and evidence-based care for patients with SUD	
**SUD Session Objectives**:	
Apply appropriate screening and diagnostic (DSM-5) tools to patients with OUD and AUD	
Identify strategies for harm reduction in patients with OUD and brief intervention in patients with AUD	
Summarize evidence-based approaches to pharmacotherapy for OUD and AUD	
Increase confidence around the diagnosis and management of patients with OUD and AUD	
Increase regard for patients with OUD and AUD	
Create an action plan for one empaneled patient with OUD and AUD	

### Evaluation methods

Needs assessment questions included demographic information, prior training in SUD, current exposure to patients with SUD, questions assessing knowledge, confidence and preparedness to diagnose and treat SUD, and attitudes towards patients with SUD. Many of the survey questions on knowledge, confidence and preparedness were replicated from Wakeman et al. 2013 [5]. The Medical Condition Regard Scale (MCRS) was used to assess attitudes towards patients with SUD [16]. 

A post-session assessment was designed for each session. It included questions duplicated from the needs assessment on knowledge, preparedness to diagnose and treat patients with OUD or AUD, and a single question from the MCRS (“Regarding patients with OUD/AUD, there is little I can do to help patients like this”), which best tied to the overall curriculum goal to “empower residents with the knowledge and skills to care for patients with substance use disorder”. Residents were also asked, using an open-ended response question, to identify one “take-away” based on each session (Online Appendix 4, 5).

Descriptive statistics were used to analyze the needs assessment and post-session evaluations.

This study was deemed exempt by the University of Chicago IRB.

## Results

Resident needs assessment (*n* = 44/105, 42% response rate) showed 86% (*n* = 37/43) characterized instruction received during residency in SUD as “none” or “too little”. While residents frequently encountered patients with SUD, only 56% (*n* = 23/41) felt prepared to diagnose and 20% (*n* = 8/41) felt prepared to treat SUD. Residents estimated an average of 25% (SD 14%) of patients on inpatient services and 14% (SD 10%) of clinic patients met criteria for SUD. 49% found patients with SUD particularly difficult to work with (*n* = 20/41, agree/strongly agree), and only 17% (*n* = 7/41, agree/strongly agree) found working with patients with SUD satisfying. 34% (*n* = 14/41) felt “there is little I can do to help” patients with SUD (not sure but probably agree/agree/strongly agree). The needs assessment questions were not required. A few surveys were incomplete which accounts for the variability in denominators. Questions that were duplicated in both the needs assessment and either the OUD or AUD session are presented with results in Table 2. The full needs assessment MCRS scores are also available (Online Appendix 6).

All residents who attended a session completed the post-session assessments. After the OUD session (*n* = 20, 100% response rate, few incomplete assessment items), all residents (*n* = 19/19) felt prepared to diagnose, and 79% (*n* = 15/19) felt prepared to treat OUD. 16% of residents (*n* = 3/19) felt “there is little I can do to help” patients with OUD. Knowledge questions about OUD diagnostic criteria and buprenorphine mechanism of action were answered correctly 67% (*n* = 12/18) and 63% (*n* = 12/19) respectively. After the OUD session, the most frequently reported take-away points included plans to screen more for OUD (*n* = 10), initiate harm reduction strategies (*n* = 5), and discuss/learn to prescribe medications for OUD (*n* = 3).

Following the AUD session (*n* = 22, 100% response rate), all residents felt prepared to diagnose (*n* = 22/22) and treat (*n* = 22/22) AUD. No residents (*n* = 0/22) felt “there is little I can do to help” patients with AUD. All residents (*n* = 22/22) answered the knowledge question about AUD pharmacotherapy correctly. The most frequently reported take-away points included screening more/differently for AUD (*n* = 8), and considering medications for AUD (*n* = 18).

For the OUD session, 52% (10/19) of residents used their own patients for the worksheet case; for the AUD session, 68% (13/19) of residents used their own patient.

**Table 2:** Comparison of select pre-curriculum needs assessment and post-session evaluation results

Domain	Item	Pre-curriculum Needs Assessment *N* = 41 % (*N*/denominator)	Post-Opioid Use Disorder Session *N* = 20 % (*N*/denominator)	Post-Alcohol Use Disorder Session *N* = 22 % (*N*/denominator)
Knowledge (correct)	DSM-5 Criteria for OUD	66 (27/41)	67 (12/18)	N/A
	Mechanism of action for buprenorphine	61 (25/41)	63 (12/19)	N/A
	AUD pharmacotherapy options	66 (27/41)	N/A	100 (22/22)
Preparedness (very or somewhat prepared)	Prepared to Diagnose	56 (23/41)	100 (19/19)	100 (22/22)
	Prepared to Treat	20 (8/41)	79 (15/19)	100 (22/22)
Attitude/Efficacy (not sure but probably agree, agree, or strongly agree)	There is little I can do to help patients like this	34 (14/41)	16 (3/19)	0 (0/22)

## Discussion

After a 2-part SUD curriculum, residents reported high levels of confidence and preparedness to diagnose and manage OUD and AUD. Residents generated take-away lessons to apply in clinical practice around SUD screening, harm reduction practices, and pharmacotherapy.

Several studies have shown that SUD curricula must effectively address knowledge gaps, and empower residents to integrate SUD recognition and management into their practices [5, 11–13, 17]. Strategies that specifically address stigma are key components given IM residents’ decreased regard for patients with SUDs, with some studies showing modest improvement in attitudes towards patients with SUDs following curricular interventions [8, 18]. This study adds to previous literature by demonstrating the effect of an SUD curriculum of shorter duration that engages residents by using their own empaneled patients as case studies. The decrease in number of residents who agreed that “there is little I can do to help” patients with SUD highlights the impact of the curriculum on attitudes towards patients with SUD and belief in their ability to act.

Using interactive worksheets and resident empaneled patients are promising strategies to deliver SUD didactics; more than 50% of residents were able to identify an empaneled patient for the session. Future work could compare curricular impact of residents using empaneled patients vs. standardized cases. Additionally, tracking whether the created action plans for empaneled patients were implemented in clinic would give more insight into the impact of the curriculum. This education strategy could also be modified for use across disciplines (OBGYN, pediatrics, surgery, emergency medicine, neurology) and learner types (medical students, fellows, faculty), and could count towards the 8-hour training requirement for DEA licensure legislated by the Medication Access and Training Expansion (MATE) Act [2]. 

Residents had high levels of comfort with diagnosing and managing AUD compared to OUD. This may be due to higher baseline comfort with AUD due to higher prevalence of alcohol use. Takeaways in the AUD session were more focused on pharmacotherapy, while in the OUD session the focus was more on screening. This divide may indicate more intense focus is needed to build resident confidence in comprehensively caring for patients with OUD, starting with screening and moving towards management.

This study is limited by occurring at a single-site, and less than half of the second and third year classes participated in the sessions. Post-session surveys were not matched to the needs assessment so direct improvements in knowledge, confidence and preparedness could not be measured. The specific impacts of the worksheet and using resident-empaneled patients (both learner receptivity and added learning value) was not separately assessed, but could be evaluated in the future. The ability to identify empaneled patients, as well as faculty time and expertise, may limit generalizability.

Developing effective SUD content for IM trainees is paramount to improve care for patients with SUD, and increasingly being required by governing bodies (e.g. ACGME, DEA). While this curriculum could be replicated exactly, a site-specific needs assessment can guide educators to identify more specific didactic focus areas (e.g. diagnosis, treatment, attitudes, self-efficacy), while maintaining the use of empaneled patients as a curricular strategy. Adaptable curricula should be created and studied for residency programs with variable levels of curricular time, existing collective knowledge, clinical experiences, and faculty expertise [9]. 

## Conclusions

Despite rising attention to SUD, there is wide variability in content coverage in IM residencies without clear ideal ways to deliver SUD content. This innovation adds a concise, adaptable, and replicable curriculum that empowers residents to integrate SUD diagnosis and management into clinical practice.

### Electronic supplementary material

Below is the link to the electronic supplementary material.

Supplementary Material 1

## Data Availability

The datasets used and/or analysed during the current study are available from the corresponding author on reasonable request.
