# Supplementary material for: A substance use disorder training curriculum for internal medicine residents using resident-empaneled patients
Source: BMC Med Educ. 2024 Apr 30;24:478. doi: 10.1186/s12909-024-05472-5 (PMC11061993; doi:10.1186/s12909-024-05472-5)
Supplement: Supplementary file 1 — Supplementary Material 1 [file 12909_2024_5472_MOESM1_ESM.docx]

**Online Appendices**

Online Appendix 1:

Safe Opioid Prescribing and Substance Use Disorder Training Curriculum

The internal medicine residency is beginning a comprehensive curriculum on safe opioid prescribing and addiction that will begin in 2019 as part of ambulatory education. To better identify and serve your needs, please fill out the attached 5-minute survey. Your feedback is very valuable.

Participation is anonymous and voluntary. Completion of the survey will indicate informed consent.

Please feel free to contact me with any questions.

Thank you in advance for completing this survey!

Mim Ari, MD

**Section A:** Demographic Information

A1: What year are you in residency?

1. PGY1
2. PGY2
3. PGY3

A2: Please indicate your gender

1. Male
2. Female
3. Other

A3: What firm are you in?

1. A
2. B
3. C

**Section B:** Medical School/Residency Experience

B1: Did your medical school offer any formal training in addiction?

1. Yes
2. No
3. Don’t know

B2: Please estimate what percentage of inpatients you care for are admitted for medical conditions directly related to substance use disorders (i.e. alcohol withdrawal, alcohol related cirrhosis, endocarditis or cellulitis related to IVDU). ______% patients admitted

B3: Please estimate what percentage of patients admitted to the inpatient service meet criteria for a substance use disorder. ______% patients admitted

B4: Please estimate what percentage of your clinic patients meet criteria for a substance use disorder. ______% clinic patients

B5: Please estimate what percentage of your clinic patients are on chronic opioid therapy. ______% clinic patients

B6: Please rate the amount of instruction in addiction you have received in residency

1. None received
2. Too little
3. Just right
4. Too much

B7: Please rate the amount of instruction in prescribing chronic opioids you have received n residency.

1. None received
2. Too little
3. Just right
4. Too much

**Section C:** Diagnosis and Management of Chronic Pain & Addiction

C1: Please rate the extent to which you feel prepared to diagnose addiction

1. Very prepared
2. Somewhat prepared
3. Somewhat unprepared
4. Very unprepared

C2: Please rate the extent to which you feel prepared to treat addiction

1. Very prepared
2. Somewhat prepared
3. Somewhat unprepared
4. Very unprepared

C3: Please rate the extent to which you feel prepared to manage patients on chronic opioid therapy

1. Very prepared
2. Somewhat prepared
3. Somewhat unprepared
4. Very unprepared

C4: To meet criteria for substance use disorder a patient must demonstrate tolerance or withdrawal

1. True
2. False
3. Don’t know

C5: Buprenorphine works as a(n)

1. Full opioid agonist
2. Partial opioid agonist
3. Opioid antagonist
4. Combination opioid agonist/antagonist
5. Don’t know

C6: Which of the following are pharmacologic treatments for alcohol use disorder?

1. Disulfiram
2. Naloxone
3. Naltrexone
4. Acamprosate
5. A&D
6. A&B
7. A, B & C
8. A, C & D
9. All of the above
10. Don’t know

C7: Genetic factors account for roughly ___ percent of an individual’s addiction vulnerability.

1. Less than 10
2. 25
3. 50
4. 80
5. Don’t know

C8: Which of the following is an indication to consider prescribing naloxone for a patient on chronic opioids?

1. History of overdose or substance use disorder
2. Concurrent benzodiazepine use
3. Opioid dose of 30 MME/day (morphine milligram equivalents)
4. A & B
5. A & C
6. A, B & C
7. Don’t know

**Section D: Attitudes and Beliefs**

| Regarding Patients with **Addiction/Substance Use Disorders** | Strongly Disagree | Disagree | Not sure but probably disagree | Not sure but probably agree | Agree | Strongly Agree |
| --- | --- | --- | --- | --- | --- | --- |
| 1. I prefer not to work with patients like this | 1 | 2 | 3 | 4 | 5 | 6 |
| 1. Patients like this irritate me | 1 | 2 | 3 | 4 | 5 | 6 |
| 1. I enjoy giving extra time to patients like this | 1 | 2 | 3 | 4 | 5 | 6 |
| 1. Patients like this are particularly difficult to work with | 1 | 2 | 3 | 4 | 5 | 6 |
| 1. Working with patients like this is satisfying | 1 | 2 | 3 | 4 | 5 | 6 |
| 1. I feel especially compassionate towards patients like this | 1 | 2 | 3 | 4 | 5 | 6 |
| 1. I wouldn’t mind getting up on call nights to care for patients like this | 1 | 2 | 3 | 4 | 5 | 6 |
| 1. I can usually find something that helps patients like this feel better | 1 | 2 | 3 | 4 | 5 | 6 |
| 1. There is little I can do to help patients like this | 1 | 2 | 3 | 4 | 5 | 6 |
| 1. Insurance plans should covers patients like this to the same degree that they cover patients with other conditions | 1 | 2 | 3 | 4 | 5 | 6 |
| 1. Treating patients like this is a waste of medical dollars | 1 | 2 | 3 | 4 | 5 | 6 |

Online Appendix 2

**A Patient with Opioid Use Disorder**

**Is this worksheet based on your PCG clinic patient?** Yes or No

1. **Chart Review:**

| 1. Visit date? |  |
| --- | --- |
| 1. Is opioid use disorder (OUD) on the problem list? |  |
| 1. Is OUD in the A&P section? |  |
| 1. Is the patient on a medication for OUD (MOUD)? |  |
| 1. Is the patient engaged in therapy or peer support (NA/SMART recovery)? |  |
| 1. Does the patient have history of overdose? |  |
| 1. Has the patient been prescribed naloxone? |  |
| 1. Does the patient have a urine drug screen? |  |
| 1. Has the patient been assessed for other substance use (alcohol, tobacco, cocaine)? |  |
| 1. Has the patient been assessed for co-occurring mental health diagnoses? |  |
| 1. In one word, how do you feel when you see this patient in clinic? |  |

1. **Apply the DSM-5 Criteria—**Based on your notes and prior interactions with the patient, attempt to fill out the DSM-5 Criteria for Opioid Use Disorder.


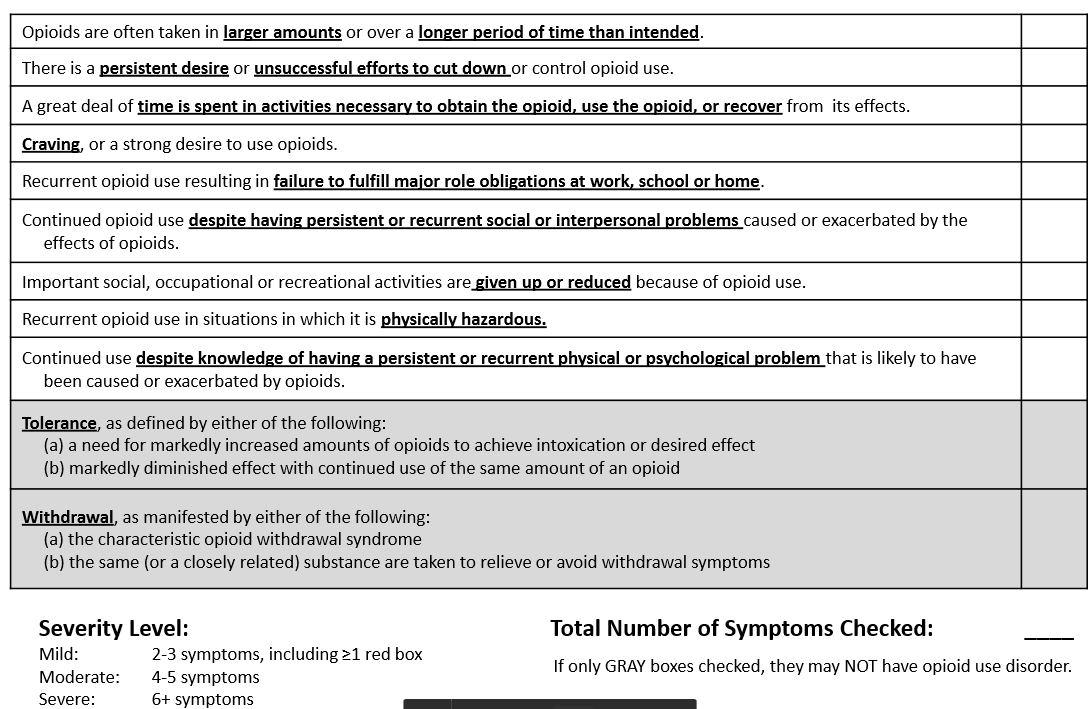


1. **MOUD:** What type of MOUD (methadone, buprenorphine, naltrexone, none) do you think would work best for this patient?
2. **Plan for Action:** Please list three things you would do next time you see this patient in clinic to better care for their opioid use disorder

1.

2.

3.

Online Appendix 3

**A Patient with Alcohol Use Disorder**

**Is this worksheet based on your PCG clinic patient?** Yes or No

1. **Chart Review:**

| 1. Visit date? |  |
| --- | --- |
| 1. Is alcohol use disorder (AUD) or unhealthy alcohol use on the problem list? |  |
| 1. Is AUD/unhealthy alcohol use in the A&P section? |  |
| 1. Is the patient on a medication for AUD (MAUD)? |  |
| 1. Is the patient engaged in therapy or peer support (AA/SMART recovery)? |  |
| 1. Does the patient have history of hospitalizations/ED visits for their alcohol use (intoxication or withdrawal)? |  |
| 1. Has the patient been assessed for other substance use (opioids, tobacco, cocaine)? |  |
| 1. Has the patient been assessed for co-occurring mental health diagnoses? |  |
| 1. In one word, how do you feel when you see this patient in clinic? |  |

1. **Apply the AUDIT or DSM-5 Criteria—**Based on your notes and prior interactions with the patient, attempt to fill out the AUDIT or DSM-5 Criteria for Alcohol Use Disorder.


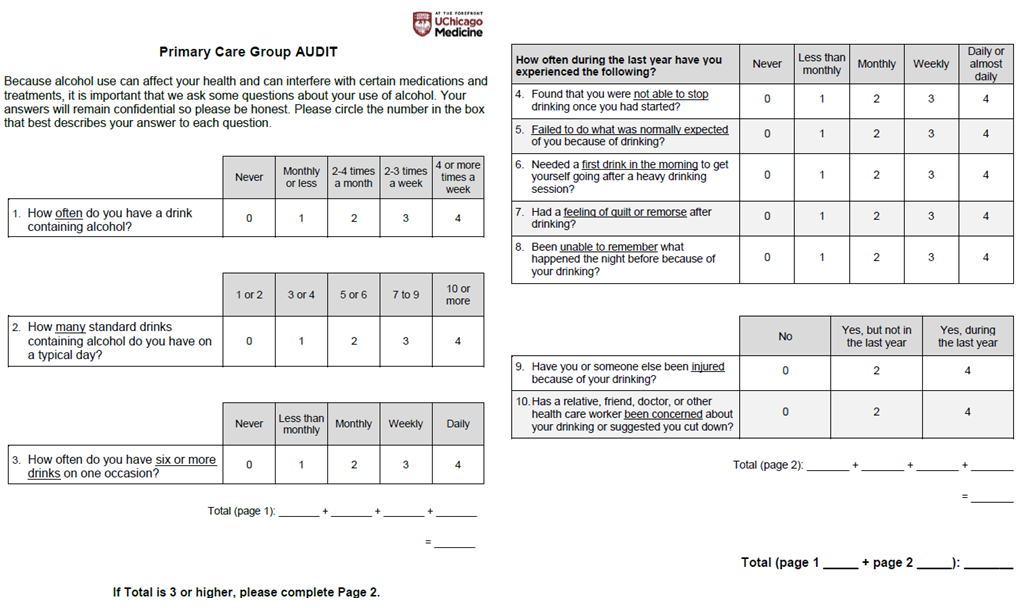


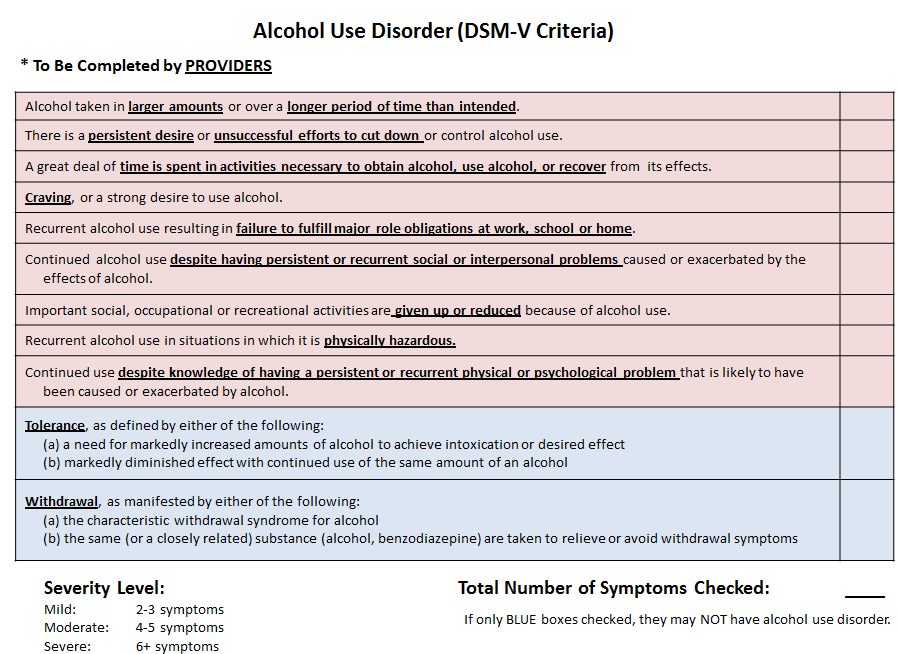


1. **MAUD:** What type of MAUD (naltrexone, acamprosate, disulfiram, none) do you think would work best for this patient?
2. **Plan for Action:** Please list three things you would do next time you see this patient in clinic to better care for their alcohol use disorder/risky alcohol use?

1.

2.

3.

Online Appendix 4

**Opioid Use Disorder Post-Session Evaluation**

1. To meet criteria for a substance use disorder a patient must demonstrate tolerance or withdrawal
   1. True
   2. False
2. Buprenorphine works as a(n)
   1. Full opioid agonist
   2. Partial opioid agonist
   3. Opioid antagonist
   4. Combination opioid agonist/antagonist
3. Please rate the extent to which you feel prepared to diagnose opioid use disorder
   1. Very prepared
   2. Somewhat prepared
   3. Somewhat unprepared
   4. Very unprepared
4. Please rate the extent to which you feel prepared to treat opioid use disorder
   1. Very prepared
   2. Somewhat prepared
   3. Somewhat unprepared
   4. Very unprepared
5. Regarding patients with opioid use disorder, there is little I can do to help patients like this
   1. Strongly Disagree
   2. Disagree
   3. Not sure but probably disagree
   4. Not sure but probably agree
   5. Agree
   6. Strongly Agree
6. **Take-away**: Please list one thing you will do differently in your practice based on this session:

**Comments/suggestions to improve this session**:

Online Appendix 5

**Alcohol Use Disorder Post-Session Evaluation:**

1. Which of the following are pharmacologic treatments for alcohol use disorder (circle all that apply)?
   1. Disulfiram
   2. Naloxone
   3. Naltrexone
   4. Acamprosate
2. Please rate the extent to which you feel prepared to diagnose alcohol use disorder
   1. Very prepared
   2. Somewhat prepared
   3. Somewhat unprepared
   4. Very unprepared
3. Please rate the extent to which you feel prepared to treat alcohol use disorder
   1. Very prepared
   2. Somewhat prepared
   3. Somewhat unprepared
   4. Very unprepared
4. Regarding patients with alcohol use disorder, there is little I can do to help patients like this
   1. Strongly Disagree
   2. Disagree
   3. Not sure but probably disagree
   4. Not sure but probably agree
   5. Agree
   6. Strongly Agree
5. **Take-away**: Please list one thing you will do differently in your practice based on this session:

**Comments/suggestions to improve this session**:

Online Appendix 6

**Medical Condition Regard Scale (MCRS) Results from the Needs Assessment (n=41)**

| **Regarding Patients with** **Addiction/Substance Use Disorders**  **%, (N)** | Strongly Disagree | Disagree | Not sure but probably disagree | Not sure but probably agree | Agree | Strongly Agree |
| --- | --- | --- | --- | --- | --- | --- |
| I prefer not to work with patients like this | 7.3 (3) | 14.6 (6) | 26.8 (11) | 31.7 (13) | 19.5 (8) | 0 (0) |
| Patients like this irritate me | 4.9 (2) | 24.4 (10) | 24.4 (10) | 36.6 (15) | 9.8 (4) | 0 (0) |
| I enjoy giving extra time to patients like this | 2.4 (1) | 34.1 (14) | 26.8 (11) | 12.2 (5) | 19.5 (8) | 4.9 (2) |
| Patients like this are particularly difficult to work with | 0 (0) | 9.8 (4) | 9.8 (4) | 31.7 (13) | 43.9 (18) | 4.9 (2) |
| Working with patients like this is satisfying | 2.4 (1) | 24.4 (10) | 19.5 (8) | 36.6 (15) | 14.6 (6) | 2.4 (1) |
| I feel especially compassionate towards patients like this | 0 (0) | 17.1 (7) | 41.5 (17) | 19.5 (8) | 19.5 (8) | 2.4 (1) |
| I wouldn’t mind getting up on call nights to care for patients like this | 4.9 (2) | 34.1 (14) | 26.8 (11) | 14.6 (6) | 19.5 (8) | 0 (0) |
| I can usually find something that helps patients like this feel better | 4.9 (2) | 46.3 (19) | 17.1 (7) | 22.0 (9) | 9.8 (4) | 0 (0) |
| There is little I can do to help patients like this | 2.4 (1) | 29.3 (12) | 34.1 (14) | 24.4 (10) | 9.8 (4) | 0 (0) |
| Insurance plans should covers patients like this to the same degree that they cover patients with other conditions | 0 (0) | 2.4 (1) | 7.3 (3) | 22.0 (9) | 48.8 (20) | 19.5 (8) |
| Treating patients like this is a waste of medical dollars | 41.5 (17) | 48.8 (20) | 4.9 (2) | 4.9 (2) | 0 (0) | 0 (0) |
